# Joint reconstruction framework of compressed sensing and nonlinear parallel imaging for dynamic cardiac magnetic resonance imaging

**DOI:** 10.1186/s12880-021-00685-2

**Published:** 2021-12-01

**Authors:** Zhanqi Hu, Cailei Zhao, Xia Zhao, Lingyu Kong, Jun Yang, Xiaoyan Wang, Jianxiang Liao, Yihang Zhou

**Affiliations:** 1 Shenzhen Children’s Hospital, Shenzhen, 518038 Guangdong China; 2 Shenzhen College of Advanced Technology, University of Chinese Academy of Sciences, Shenzhen, 518055 Guangdong China; 3 School of Physics and Electronic Engineering, Yuxi Normal University, Yuxi, 653100 Yunnan China; 4 Hong Kong Sanatorium and Hospital, 5 A Kung Ngam Village Road, Shau Kei Wan, Hong Kong, China

**Keywords:** Dynamic cardiac MRI, Compressed sensing, Parallel imaging, Nonlinear GRAPPA

## Abstract

Compressed Sensing (CS) and parallel imaging are two promising techniques that accelerate the MRI acquisition process. Combining these two techniques is of great interest due to the complementary information used in each. In this study, we proposed a novel reconstruction framework that effectively combined compressed sensing and nonlinear parallel imaging technique for dynamic cardiac imaging. Specifically, the proposed method decouples the reconstruction process into two sequential steps: In the first step, a series of aliased dynamic images were reconstructed from the highly undersampled k-space data using compressed sensing; In the second step, nonlinear parallel imaging technique, i.e. nonlinear GRAPPA, was utilized to reconstruct the original dynamic images from the reconstructed k-space data obtained from the first step. In addition, we also proposed a tailored k-space down-sampling scheme that satisfies both the incoherent undersampling requirement for CS and the structured undersampling requirement for nonlinear parallel imaging. The proposed method was validated using four in vivo experiments of dynamic cardiac cine MRI with retrospective undersampling. Experimental results showed that the proposed method is superior at reducing aliasing artifacts and preserving the spatial details and temporal variations, compared with the competing k-t FOCUSS and k-t FOCUSS with sensitivity encoding methods, with the same numbers of measurements.

## Introduction

Dynamic Magnetic resonance imaging (dMRI) is an important medical imaging modality for the diagnosis of cardiovascular diseases. Due to its excellent tissue contrast, MRI can effectively evaluate cardiac functions and vascular abnormalities by acquiring a time series of images at a high frame rate during cardiac motion. To obtain an artifact-free image series using conventional Fourier reconstruction, the Nyquist sampling requirement must be satisfied in both spatial and temporal directions. However, due to the low data acquisition speed, dynamic MRI does not always meet this criterion. Hence, dynamic MRI often suffers from aliasing or motion artifacts. The acquisition speed is hence of primary importance for achieving high spatial-temporal resolution in dynamic MRI applications. A range of techniques have been developed to reconstruct a high-quality image series from the undersampled MRI data by exploiting spatial and/or temporal correlations in the dynamic image series. These spatial and temporal correlations render it feasible to estimate the missing data from undersampled measurements. Typical methods for the reconstruction of undersampled single-coil measurements include RIGR [1], keyhole [2], view-sharing [3], UNFOLD [4], Partially Separable Function (PS) [5–7], Kalman filter [8], PARADIGM [9], k-t BLAST [10] and so on.

Two promising techniques for speeding up acquisition are parallel imaging and compressed sensing, which downsample the k-space below the Nyquist rate. Parallel imaging [11–14] uses information from multiple coil signals to estimate the unacquired k-space data, such as the classical SENSE [12] and GRAPPA [13]. Theoretically, the maximum acceleration rate of parallel imaging can be up to the number of physical channels under ideal conditions. However, noise and imperfect coil geometry limit the achievement to the maximum acceleration in practice. Compressed sensing (CS) [15–17] exploits the sparseness of the signal in a certain domain as the prior constraint and recovers the accurate signal using nonlinear optimization algorithms. The success of applying CS to dynamic cardiac MRI greatly accelerates the acquisition process [18–26]. Such success is based on two important properties of the dynamic cardiac images: firstly, the dynamic cardiac images exhibit strong correlations between frames which guarantee the sparse representation of the sequence in a specific transform domain; secondly, the sampling pattern can be easily designed to satisfy the incoherence requirement of CS theory.

Since the sampling scheme of CS and parallel imaging exploit complementary redundancy information in MRI, it is of great interest to combine these two techniques so that a higher reduction factor could be achieved. Several studies attempted to combine parallel MRI (pMRI) and CS for static imaging [27–39]. Conventionally, the multi-coil information was incorporated into the CS framework by replacing the Fourier encoding with sensitivity encoding in the data consistency term [25, 27, 35], together with any sparsifying transform such as the low-rank model [31, 32], the dictionary learning [33, 34]. In addition, the recent development of deep learning techniques also attempted to combine the CS and pMRI frameworks into various cardiac MRI applications [37–39]. However, few studies explored how to efficiently combine CS and pMRI approaches to maximize accelerations, given the different sample requirements of the two approaches.

In this paper, we proposed a novel reconstruction framework that efficiently combines compressed sensing and non-linear parallel imaging technique to accelerate dynamic cardiac imaging, extending results in our conference papers [40, 41]. The proposed framework decouples the reconstruction process into two sequential steps. In the first step, the highly undersampled k-space data is used to recreate a series of aliased dynamic cardiac images using a CS approach; In the second step, nonlinear GRAPPA [42–44] was used to remove the aliasing artifacts and recover the desired dynamic cardiac cine images. In addition, the sampling pattern for each step is also designed accordingly, allowing for simultaneous satisfaction of the incoherent undersampling need for CS and the structured undersampling requirement for parallel imaging. The proposed method was validated on four in vivo human cardiac cine MR datasets. Experimental results indicated that the proposed joint reconstruction framework could effectively combine the CS and parallel imaging to improve the reconstruction quality of dynamic cardiac MRI, comparing to the conventional methods.

## Theory and method

In the data acquisition process, the k-t space is undersampled by taking a random subset of the already uniformly undersampled k-space data at each time point. Mathematically, let ${\mathbf{M}}_{{\text{u}}}$ represent the uniform undersampling operation, ${\mathbf{M}}_{{\text{r}}}$ represents the operation of taking a random subset in k-space. The data acquisition process can be expressed by:

1 $${\mathbf{d}}_{{{\text{u}},{\text{r}}}} = {\mathbf{M}}_{{\text{r}}} {\mathbf{M}}_{{\text{u}}} {\mathbf{d}}_{{{\text{full}}}}$$

where ${\mathbf{d}}_{{{\text{u}},{\text{r}}}}$ is the acquired data, and ${\mathbf{d}}_{{{\text{full}}}}$ is the fully sampled reference data in k-t space. The uniform undersampling has a reduction factor of *R*_1_, and the random subset has a reduction factor of *R*_2_ for the CS requirement. As a result, the total acceleration is *R*_1_ × *R*_2_. For all time frames, additional auto calibration signals (ACS) are also acquired at the center k-space.

Based on the decoupled undersampling operations ${\mathbf{M}}_{{\text{u}}}$ and ${\mathbf{M}}_{{\text{r}}}$, the reconstruction is carried out in two sequential steps. The CS reconstruction framework is utilized in the first stage, with the goal of reconstructing a uniformly undersampled k-space by “inverting” the operation ${\mathbf{M}}_{{\text{r}}}$. Let the aliased image series to be reconstructed in the (x-f) domain is represented by ${{\varvec{\uprho}}}_{{\text{u}}}$. The data consistency term is then given by

2 $${\mathbf{d}}_{{\text{u}},{\text{r}}} = {\bf{F}}{{\varvec{\uprho}}}_{\text{u}}$$

where ${\mathbf{F}}$ is the Fourier transform along both *x* and *f* directions. The reconstruction of the signal ${{\varvec{\uprho}}}_{{\text{u}}}$ in *x-f* domain can be modeled as a truncated *ℓ*_1_ minimization problem

3 $$\mathop {\min }\limits_{{{{\varvec{\uprho}}}_{{\text{u}}} }} \|{{\varvec{\uprho}}}_{{{\text{u}}}}\|_{1} \quad s.t.\quad \|{\mathbf{d}}_{{\text{u,r}}} - {\mathbf{F}}{{{\varvec{\uprho}}}_{{\text{u}}}}\|_{2} \le \varepsilon$$

Here we use the k-t FOCUSS [21] algorithm to reconstruct the aliased image sequence in the temporal-frequency (*x-f*) domain, although other methods are also applicable.

The solution to Eq. (3) is computed by iteratively solving a reweighted *ℓ*2 minimization problem defined as

$$\text{find } {{\varvec{\uprho}}}_{{\mathbf{u}}} = {\mathbf{Wq}}$$

such that **q** is the solution to

4 $$\mathop {\min }\limits_{{\mathbf{q}}} \|{\mathbf{q}}\|_{2} \quad\text{s.t.}\quad\|{{\mathbf{d}}_{{{\text{u}},{\text{r}}}}} - {\mathbf{FWq}}\|_{2} \le {\varepsilon}$$

where **W** is a diagonal weighting matrix that is updated iteratively. Equation (4) can be converted into an unconstrained optimization problem by introducing the Lagrangian multiplier *λ*,

5 $$\mathop {\min }\limits_{{\mathbf{q}}} \|{{\mathbf{d}}_{{{\text{u}},{\text{r}}}}} - {\mathbf{FWq}}\|_{2}^{2} + \lambda\|{\mathbf{q}}\|_{2}^{2}$$

which has a closed-form solution

6 $${\mathbf{q}} = {\mathbf{W}}^{{\mathbf{H}}} {\mathbf{F}}^{{\mathbf{H}}} \left( {{\mathbf{FWW}}^{{\mathbf{H}}} {\mathbf{F}}^{{\mathbf{H}}} + \lambda{\mathbf{I}}} \right)^{ - 1} {\mathbf{d}}$$

Conjugate gradient is used to solve Eq. (6) to avoid the large matrix inversion. So the *x-f* image is given by

7 $${{{\varvec{\uprho}}}_{{\text{u}}}} = {\mathbf{Wq}} = {\mathbf{WW}}^{{\mathbf{H}}} {\mathbf{F}}^{{\mathbf{H}}} \left( {{\mathbf{FWW}}^{{\mathbf{H}}} {\mathbf{F}}^{{\mathbf{H}}} + \lambda{\mathbf{I}}} \right)^{ - 1} {\mathbf{d}}$$

Specifically, in the *l*-th iteration, the diagonal elements of the matrix ${\mathbf{W}}^{{\left( {l} \right)}}$ are the square root of the absolute value of the solution ${{\varvec{\uprho}}}_{{\text{u}}}^{{\left( {{l} - 1} \right)}}$ from the previous iteration,

8 $${\mathbf{W}}^{(l)} = \left( {\begin{array}{*{20}c} {\left| {{{\varvec{\uprho}}}_{1}^{(l - 1)} } \right|^{0.5} } & 0 & \cdots & 0 \\ 0 & {\left| {{{\varvec{\uprho}}}_{2}^{(l - 1)} } \right|^{0.5} } & \cdots & 0 \\ \vdots & \vdots & \ddots & \vdots \\ 0 & 0 & \cdots & {\left| {{{\varvec{\uprho}}}_{n}^{(l - 1)} } \right|^{0.5} } \\ \end{array} } \right),$$

where ${{\varvec{\uprho}}}_{{n}}^{{\left( {{l} - 1} \right)}}$ is the nth element of ${{\varvec{\uprho}}}_{{\text{u}}}^{{\left( {{l} - 1} \right)}}$. The reconstructed ${{\varvec{\uprho}}}_{{\text{u}}}$ is then Fourier transformed along both spatial and temporal frequency directions to obtain the uniformly undersampled data in k-t space.

The missing k-space lines in the reconstructed uniformly undersampled data in k-t space from the CS step are then further reconstructed using parallel imaging techniques. To avoid the computation of sensitivity maps and to minimize the error/noise amplification, here we employed nonlinear GRAPPA technique [42]. However, it should be noted that any advanced parallel imaging methods could also be easily integrated into the proposed framework. The pre-acquired ACS lines are used to normalize the reconstructed k-space to correct any mismatch. Specifically, we assume the reconstructed and the acquired data differ by a scaling complex constant:

9 $${\mathbf{d}}_{{{\text{recon}}}} = {{\beta}}{\mathbf{d}}_{{{\text{acq}}}}$$

where *β* is the scaling factor. The k-space for each frame is then scaled based on the overlapping locations of reconstructed and acquired data.

**Fig. 1:**
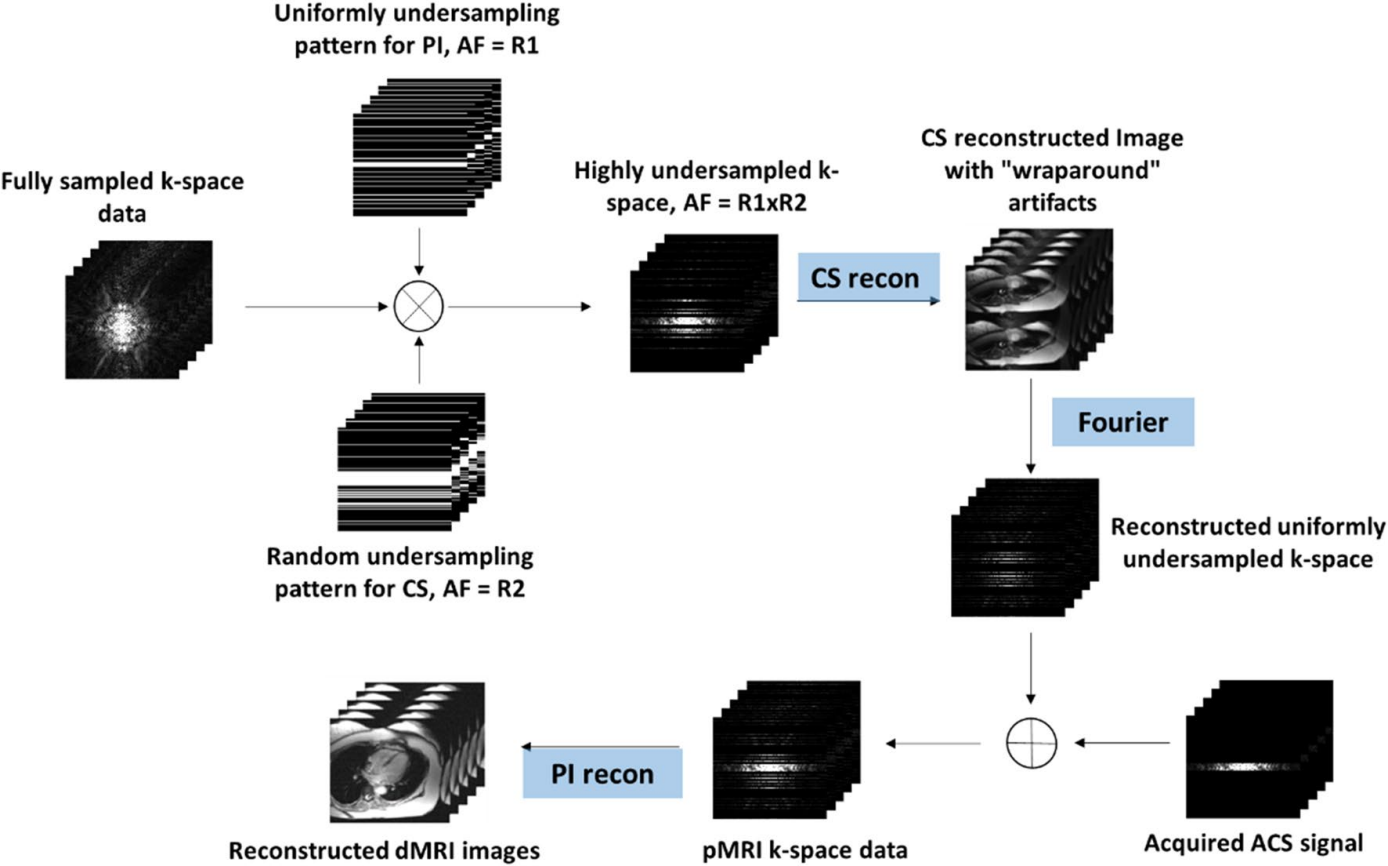
Reconstruction flowchart of the proposed method

Nonlinear GRAPPA [42–44] is conducted after normalization. In nonlinear GRAPPA, the missing data is represented by a nonlinear combination of the acquired data. Here we apply a truncated second-order polynomial whose efficiency and effectiveness have been demonstrated in [42–44]. The missing k-space signal *S*_*j*_ in ${\mathbf{d}}_{{{\text{recon}}}}$ is obtained by

10 $$\begin{aligned} &S_{j} \left( {k_{y} + r\Delta k_{y} ,k_{x} } \right) = w_{j,r}^{\left( 0 \right)} \times 1 \\ &\quad+ \sum\limits_{l = 1}^{L} {\sum\limits_{{b = B_{1} }}^{{B_{2} }} {\sum\limits_{{h = H_{1} }}^{{H_{2} }} {w_{j,r}^{\left( 1 \right)} \left( {l,b,h} \right) \times S_{l} \left( {k_{y} + bR\Delta k_{y} ,k_{x} + h\Delta k_{x} } \right)} } } \\ &\quad+ \sum\limits_{l = 1}^{L} {\sum\limits_{{b = B_{1} }}^{{B_{2} }} {\sum\limits_{{h = H_{1} }}^{{H_{2} }} {w_{j,r}^{{\left( {2,0} \right)}} \left( {l,b,h} \right) \times S_{l}^{2} \left( {k_{y} + bR\Delta k_{y} ,k_{x} + h\Delta k_{x} } \right)} } } \\ &\quad+ \sum\limits_{l = 1}^{L} {\sum\limits_{{b = B_{1} }}^{{B_{2} }} {\sum\limits_{{h = H_{1} }}^{{H_{2} - 1}} \begin{aligned} &w_{j,r}^{{\left( {2,1} \right)}} \left( {l,b,h} \right) \times S_{l} \left( {k_{y} + bR\Delta k_{y} ,k_{x} + h\Delta k_{x} } \right) \hfill \\ &\times S_{l} \left( {k_{y} + bR\Delta k_{y} ,k_{x} + (h + 1)\Delta k_{x} } \right) \hfill \\ \end{aligned} } } \\ &\quad+ \sum\limits_{l = 1}^{L} {\sum\limits_{{b = B_{1} }}^{{B_{2} }} {\sum\limits_{{h = H_{1} }}^{{H_{2} - 2}} \begin{aligned} &w_{j,r}^{{\left( {2,2} \right)}} \left( {l,b,h} \right) \times S_{l} \left( {k_{y} + bR\Delta k_{y} ,k_{x} + h\Delta k_{x} } \right) \hfill \\ &\times S_{l} \left( {k_{y} + bR\Delta k_{y} ,k_{x} + (h + 2)\Delta k_{x} } \right), \hfill \\ \end{aligned} } } \\ \end{aligned}$$

where *S*_*l*_ represents the acquired k-space data, *w* is the coefficient set, *R* is the outer reduction factor, *j* is the target coil, *l* counts all coils, *b* and *h* transverse the acquired neighboring k-space data in *k*_*y*_ and *k*_*x*_ directions respectively, and *k*_*x*_ and *k*_*y*_ represent the coordinates along the frequency- and phase-encoding directions, respectively. The final image is obtained by combining images from all coils using root of sum-of-squares after the missing k-space data for all coils has been recovered. The proposed framework is demonstrated in Fig. 1.

Validation of the proposed method was performed on four sets of cardiac cine data from dynamic MRI, each covering a complete cardiac cycle. The imaging parameters were shown in Table 1. Informed consent was obtained from all volunteers in accordance with the institutional review board policy. The fully sampled data were uniformly undersampled with a reduction factor of *R*_1_ and furthermore randomly undersampled with a reduction factor of *R*_2_ using a zero-mean random Gaussian distribution whose density tapers off toward the outer *k*-space retrospectively.

**Table 1:** Data acquisition parameters for imaging experiments

	Data 1	Data 2	Data 3	Data 4
Scanner	3T Siemens	3T Siemens	3T Siemens	3T Siemens
Sequence	SSFP	2D true FISP	SSFP	SSFP
Flip angle	44	50	50	44
Echo time/repetition time (ms)	1.5/3.0	1.87/29.9	1.89/56.6	1.22/42.5
Matrix size (FE × PE × Frame × Coil)	166 × 130 × 15 × 5	256 × 216 × 20 × 4	256 × 224 × 14 × 4	304 × 165 × 26 × 12

The proposed method, k-t FOCUSS [24] and k-t FOCUSS with sensitivity encoding [20] were used to reconstruct the desired image sequence. The code for k-t FOCUSS [24] was obtained from http://bisp.kaist.ac.kr. Same net reduction factor was used for all methods. For the reconstruction using k-t FOCUSS, images were reconstructed for each coil separately and then combined using square-root of sum-of-squares. The center k-space was fully sampled to estimate the low-resolution image for FOCUSS algorithm, to estimate the sensitivity map for k-t FOCUSS SENSE and the Auto Calibration Data (ACS) for the proposed method. The net reduction factor *R* is defined as,

11 $${R} = \frac{{\text{number of phase encoding lines}}}{{\frac{{\text{number of PE lines}}}{{{{R}}_{1} \times {{R}}_{2} }} + \text{fully sampled center phase encoding lines}}}.$$

**Fig. 2:**
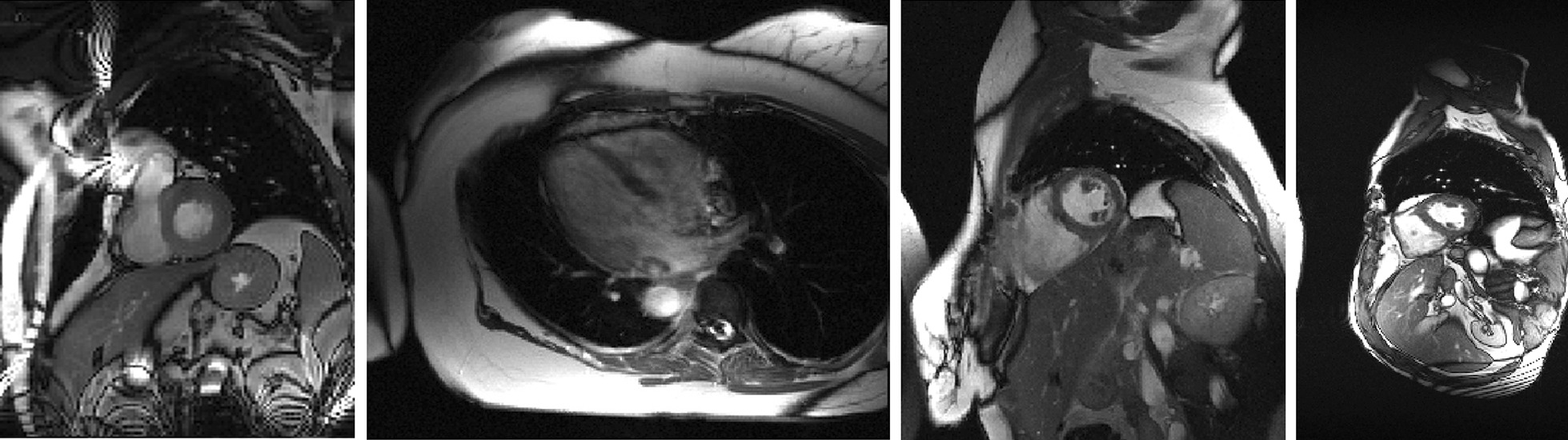
A representative image frame reconstructed from the fully sampled data for all four datasets

The images reconstructed from the full k-t data were used as the reference for comparison. All image data presented in Fig. 2 were zero-padded, normalized, and displayed on the same scale, representing the original FOV. All the computations were carried out on a workstation with Intel i7-3770 3.40 GHz CPU and 64 GB RAM running MATLAB 2019a (Mathworks, Natick, MA).

## Results

### Image quality assessment of proposed framework

**Fig. 3:**
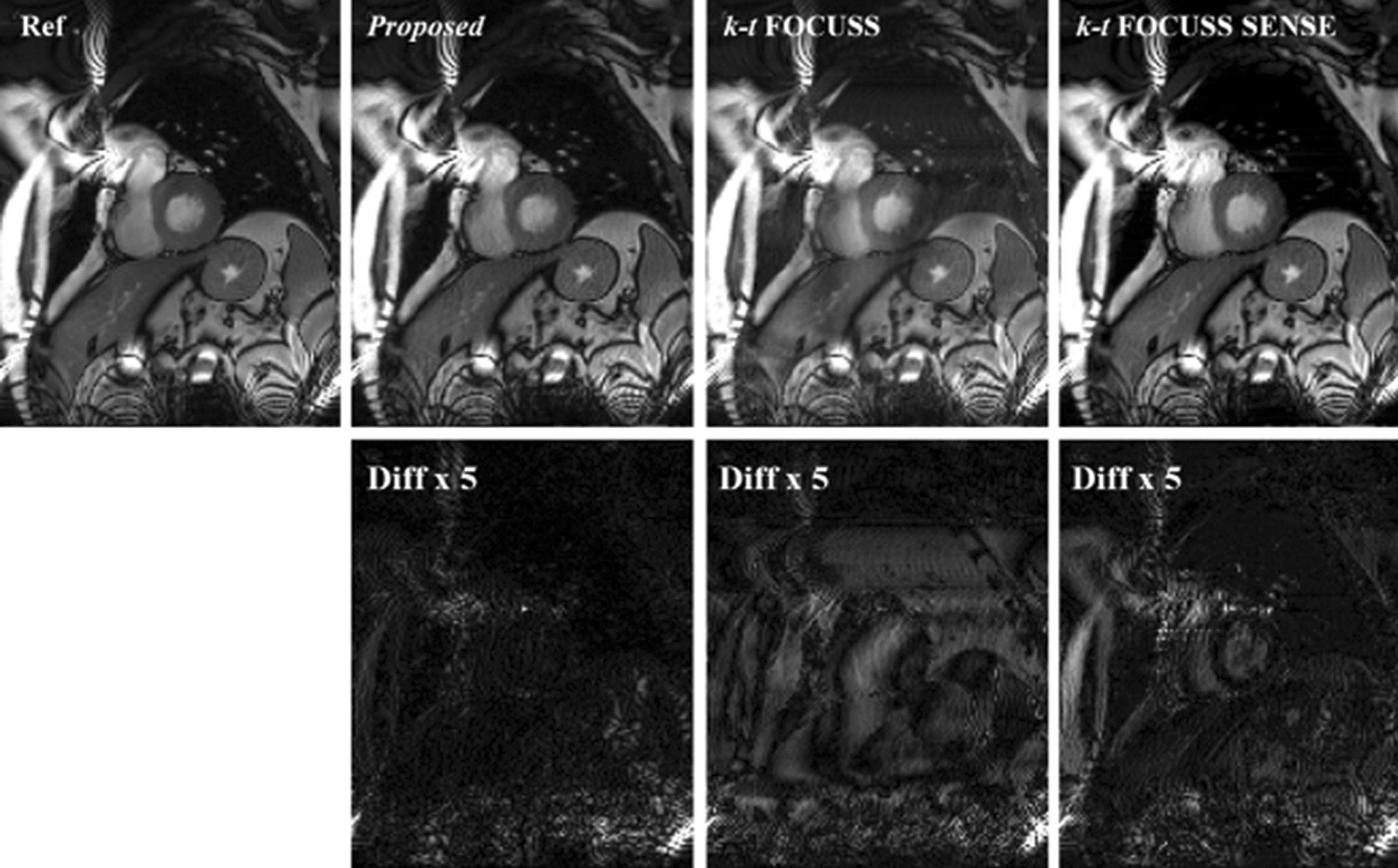
Reconstructions of the 6th frame of Data 1 using the proposed method (second column), k-t FOCUSS (third column) and k-t FOCUSS SENSE (fourth column). Center 32 phase encoding lines were fully sampled. For the proposed method, the acceleration combination was 3 for CS and 2 for Nonlinear GRAPPA. The net reduction factor was 2.89 for all methods

**Fig. 4:**
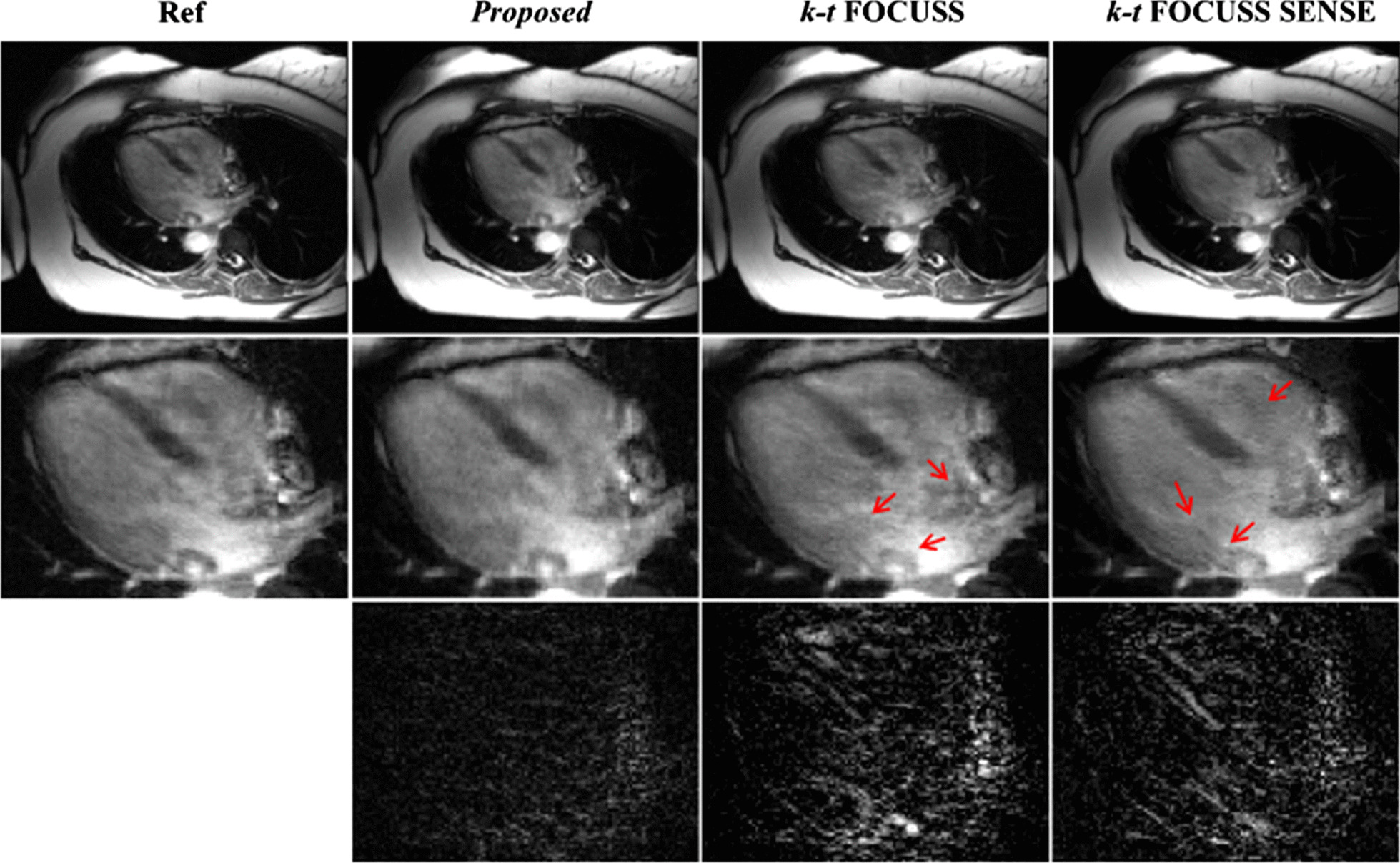
Reconstructions of the 16th frame of Data 2 using the proposed method (second column), k-t FOCUSS (third column) and k-t FOCUSS SENSE (fourth column). Center 36 phase encoding lines were fully sampled. For the proposed method, the acceleration combination was 4 for CS and 2 for Nonlinear GRAPPA. The net reduction factor was 4.11 for all methods

Figure 3 shows the reconstruction result and the corresponding error images compare with the reference at the sixth frame of the first dataset. The difference images were scaled appropriately to highlight the differences between the reconstructions and the reference images. For the proposed method, the reduction factor was 2 for parallel imaging and 3 for CS. For k-t FOCUSS [24] and k-t FOCUSS SENSE [20], the outer reduction factor was 6. The center 32 phase encoding lines were fully sampled as the ACS data and estimating the coil sensitivity, which makes the net reduction factor *R* = 2.89 for all methods. It is seen in Fig. 3 that the reconstruction using k-t FOCUSS [24] presented aliasing artifacts along the undersampled phase encoding direction. By incorporating sensitivity encoding, the aliasing artifacts were suppressed but presented noise in both cardiac region and background as indicated by arrowheads. The advantage of proposed methods on suppressing aliasing and noise artifacts can be better appreciated in the difference images. Figure 4 shows the reconstruction result of the 16th frame of the second dataset at a net reduction *R* = 4.11. For the proposed method, the acceleration combination was 4 for CS and 2 for Nonlinear GRAPPA. Center 36 phase encoding lines were fully sampled. The results of data 2 lead to the same conclusion that the proposed method can suppress more aliasing artifacts and preserve more details than either k-t FOCUSS or k-t FOCUSS SENSE. It is worth noting that although adding sensitivity encoding could significantly remove the aliasing artifacts, it over-smoothed the image, resulted in a loss of details in the cardiac region. It was mainly due to the inaccurate estimation of the sensitivity map.

**Fig. 5:**
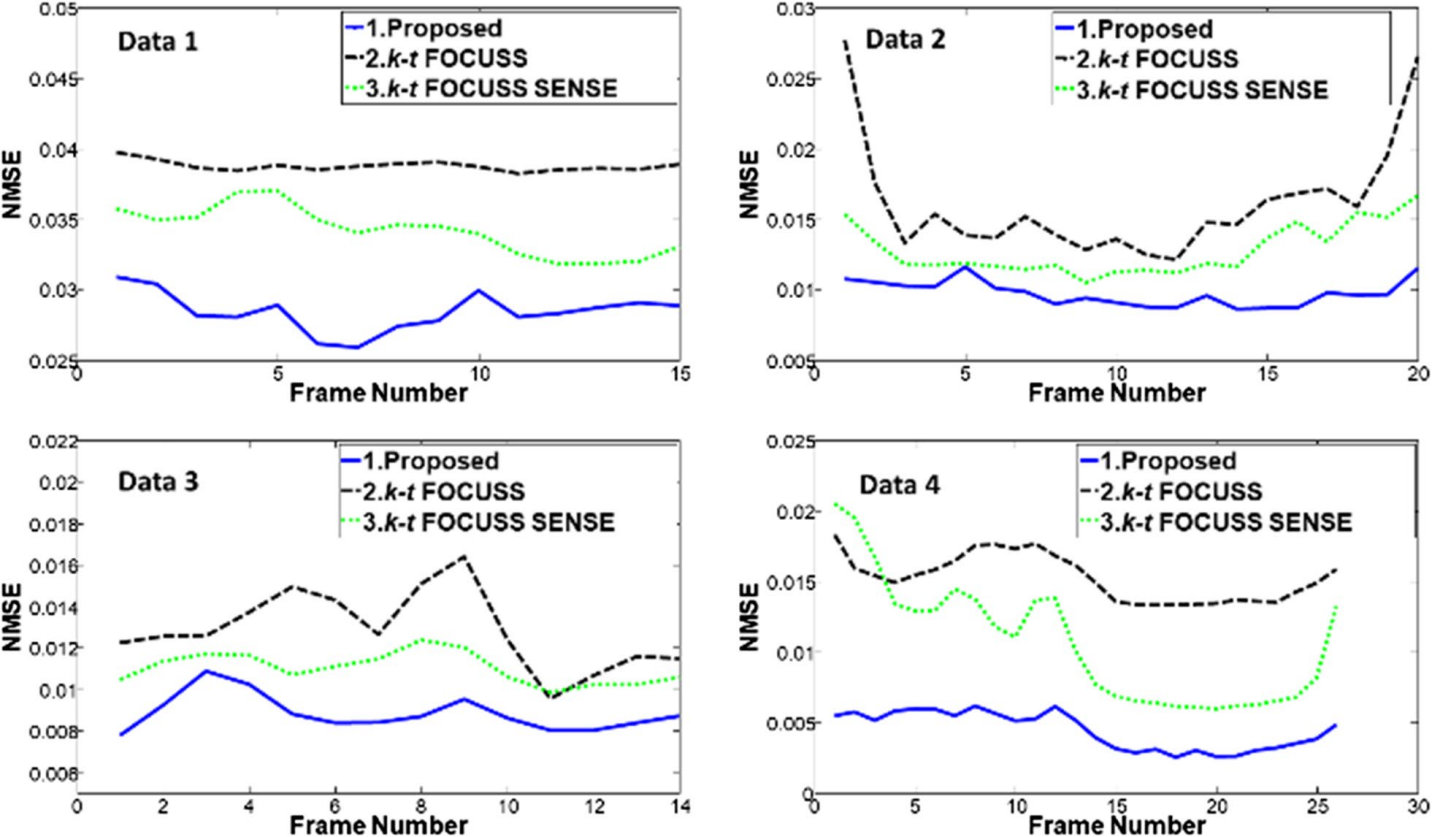
Frame-by-frame plots of NMSE in the ROI for the proposed method, k-t FOCUSS, and k-t FOCUSS with SENSE with net reduction factor R = 2.89 for the Data 1, R = 4.11 for the Data 2, R = 4.14 for the Data 3, and R = 3.6 for the Data 4. The blue solid lines are for the proposed method, green dotted lines for the k-t FOCUSS with SENSE, and black dashed lines for the k-t FOCUSS

To quantitatively evaluate the performance of the proposed method, the normalized mean-square error (NMSE) of the region of interest (ROI) between the reconstruction and the reference were calculated and plotted as a function of time in Fig. 5. The NMSE was calculated by

12 $$\text{NMSE}\left( {{\mathbf{I}}_{ref} ,{\mathbf{I}}_{recon} } \right) = \frac{{\|{\mathbf{I}}_{ref} - {\mathbf{I}}_{recon}\|_{2}^{2}}}{{\|{\mathbf{I}}_{ref}\|_{2}^{2} }}.$$

It can be seen that the proposed method has a lower NMSE comparing to the other two competing methods at all frames.

**Fig. 6:**
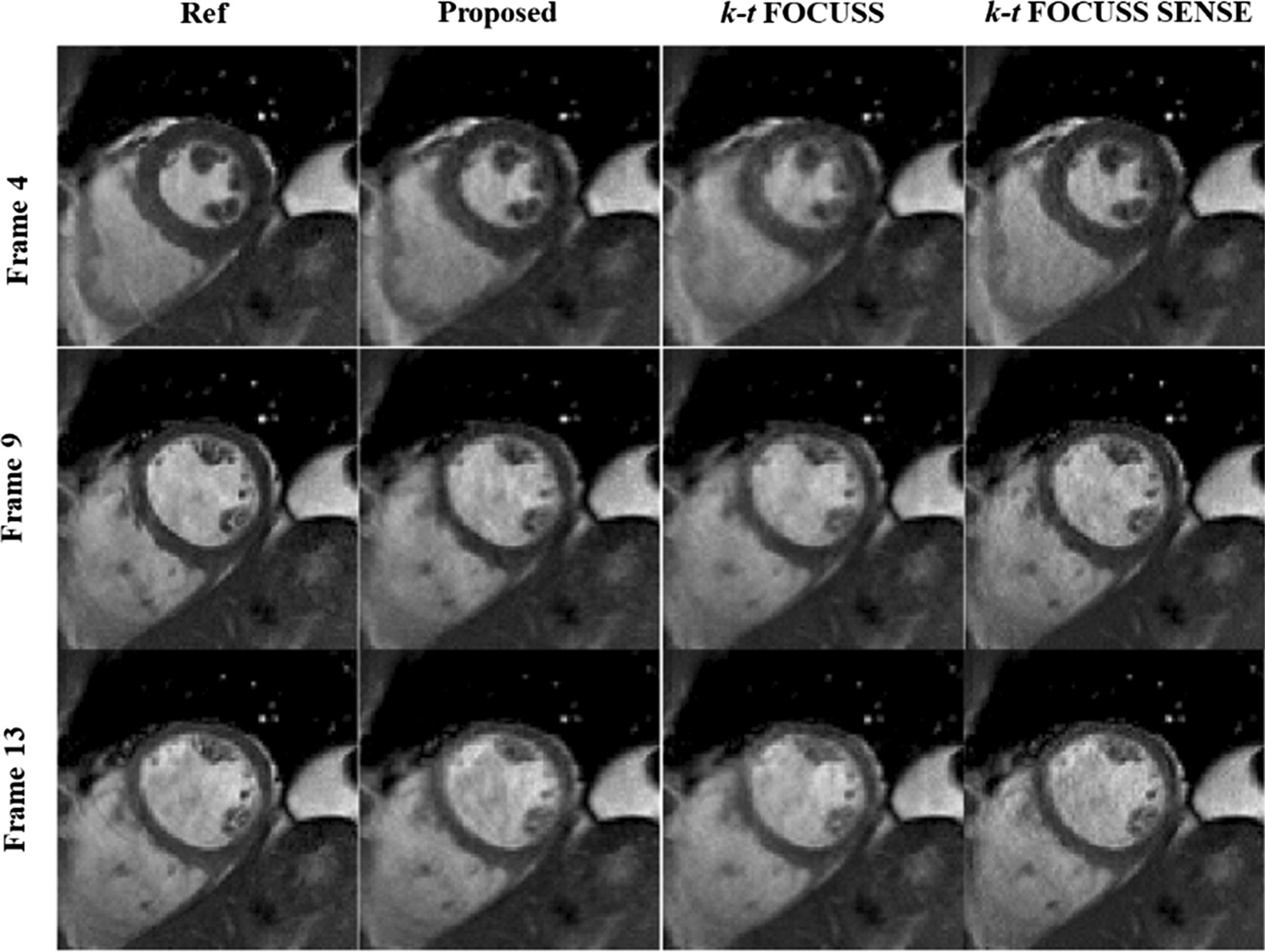
Region of interest of reference (first column), the proposed method (second column), k-*t* FOCUSS (third column) and k-*t* FOCUSS with SENSE (fourth column) of data 3. Center 38 phase encoding lines were fully sampled. For the proposed method, the acceleration combination was 4 for CS and 3 for Nonlinear GRAPPA. The net reduction factor was 4.14 for all methods

**Fig. 7:**
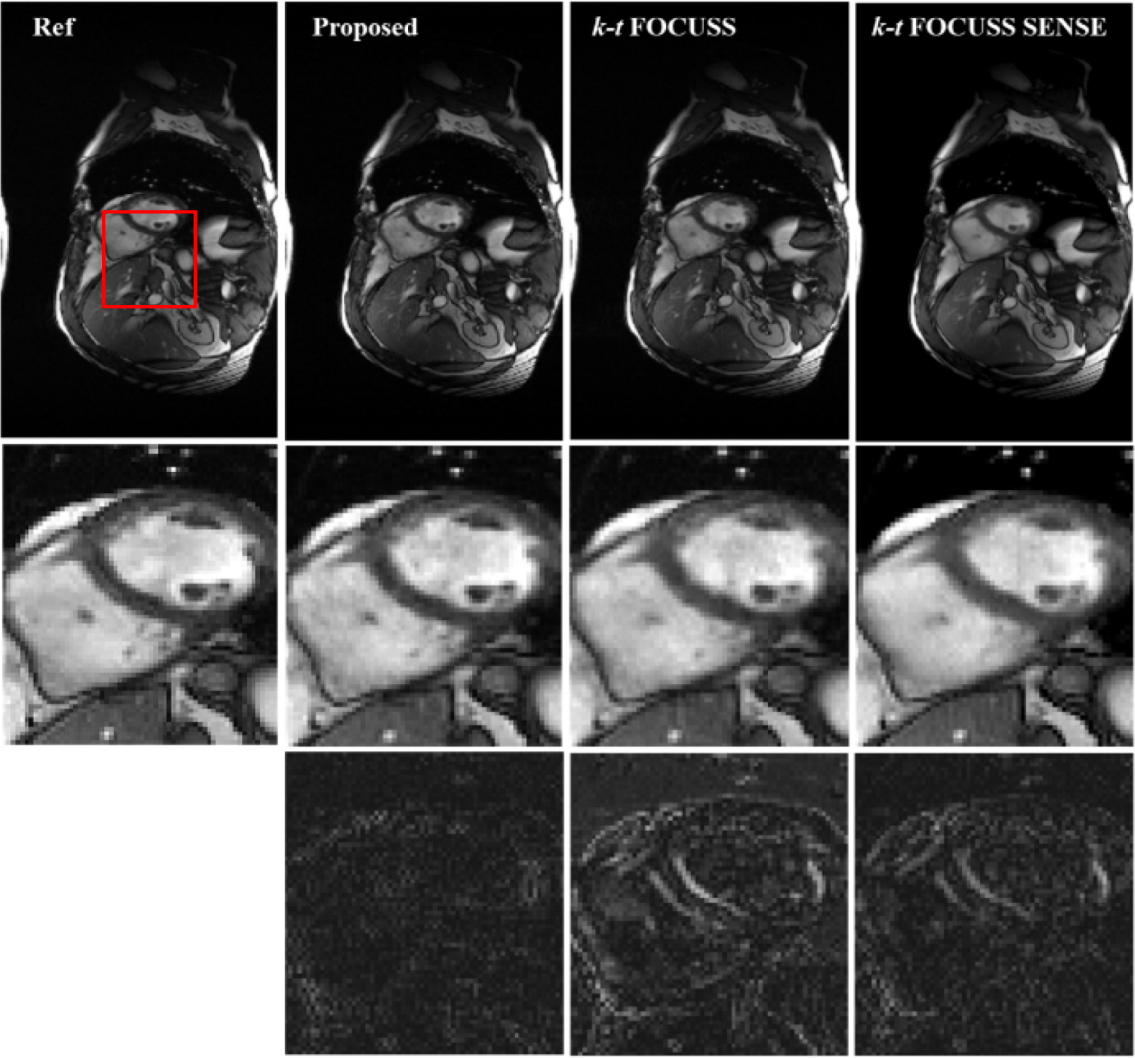
Reference (first column), the proposed method (second column), k-*t* FOCUSS (third column) and k-*t* FOCUSS with SENSE (fourth column) of data 4. Center 32 phase encoding lines were fully sampled. For the proposed method, the acceleration combination was 6 for CS and 2 for Nonlinear GRAPPA. The net reduction factor was 3.6 for all methods

To further evaluate the performance of the proposed method in clinical diagnosis, the heart region with the most dynamic motion were considered as the region of interest and zoomed-in for comparison for all methods in the third and fourth dataset. Figure 6 shows the reconstruction in the heart region comparison from different frames of the third dataset at a net reduction factor of 4.14. The acceleration combination of proposed method was 4 for CS and 3 for nonlinear GRAPPA [42–44]. The center 38 phase encoding lines were fully sampled. It can be seen that k-t FOCUSS [24] presented blurring on the image and loses details. By incorporating sensitivity encoding, k-t FOCUSS SENSE improved the blurring but exhibited noise-like artifact. In comparison, the proposed method evidently removed the blurring and the noise. Figure 7 shows the comparison between the proposed method with other three methods with Data 4. It is also suggested that the proposed method is superior at removing motion and aliasing artifact, especially at the cardiac region than the competing methods.

**Fig. 8:**
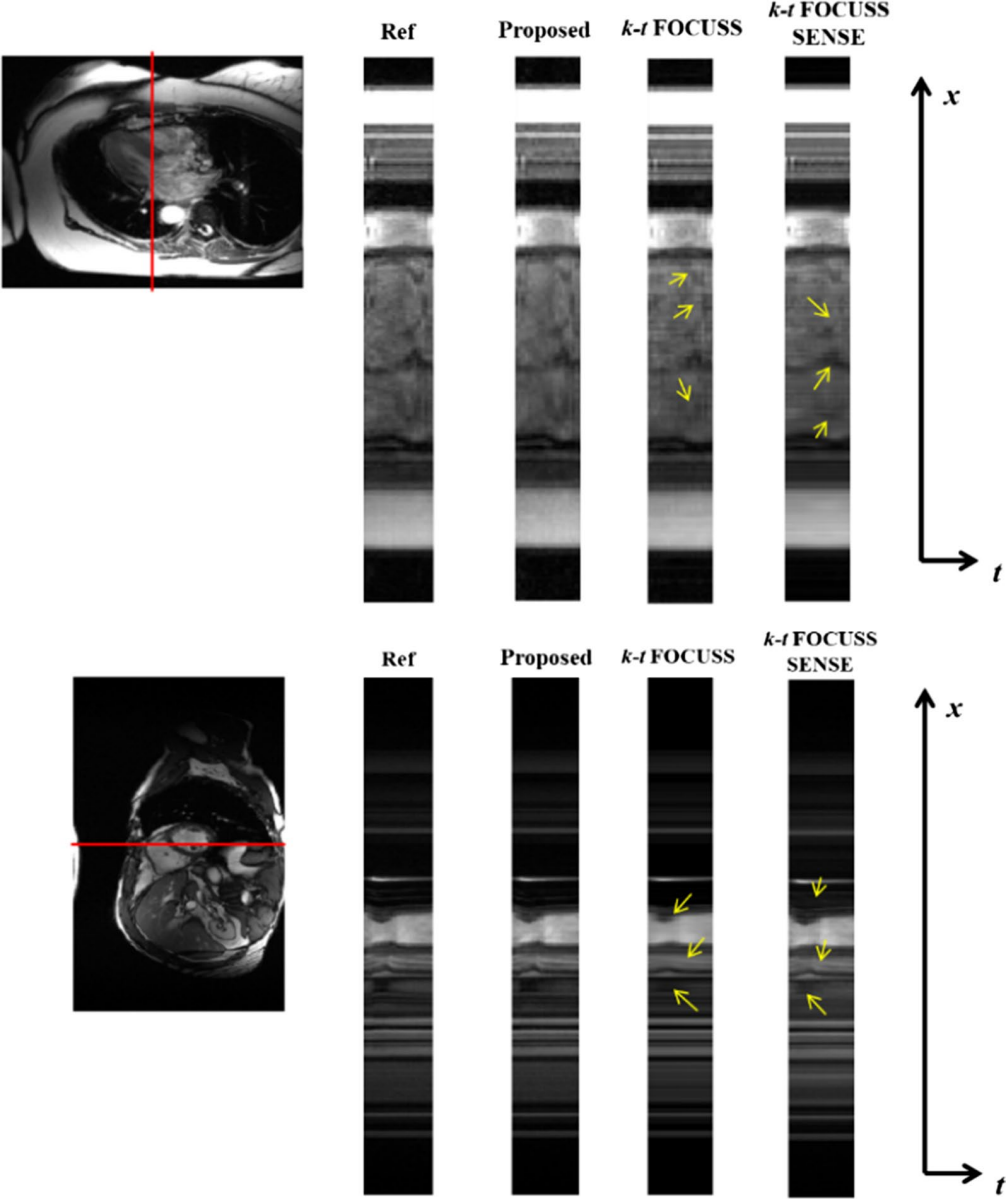
The temporal profiles in x-t plane of the different reconstruction methods, the proposed method, k-t FOCUSS and k-t FOCUSS with SENSE, for the Data 2 with net reduction factor of *R* = 4.11 and data 4 with net reduction factor of *R* = 3.6 (bottom row)

The ability to capture the temporal variation is another important criterion to evaluate the performance of dynamic reconstruction methods. The temporal profiles of the second data with a net reduction factor of *R* = 4.11 and Data 4 with a net reduction factor of *R* = 3.6 were shown in Fig. 8. It is seen that the k-t FOCUSS method [24] smoothed out the rapid temporal changes, while k-t FOCUSS SENSE [20] suffered from loss of contrast. In comparison, the proposed method preserved most of the temporal variations, especially in regions indicated by the arrowheads.

### Choice of acceleration factor combination for CS and PI

**Fig. 9:**
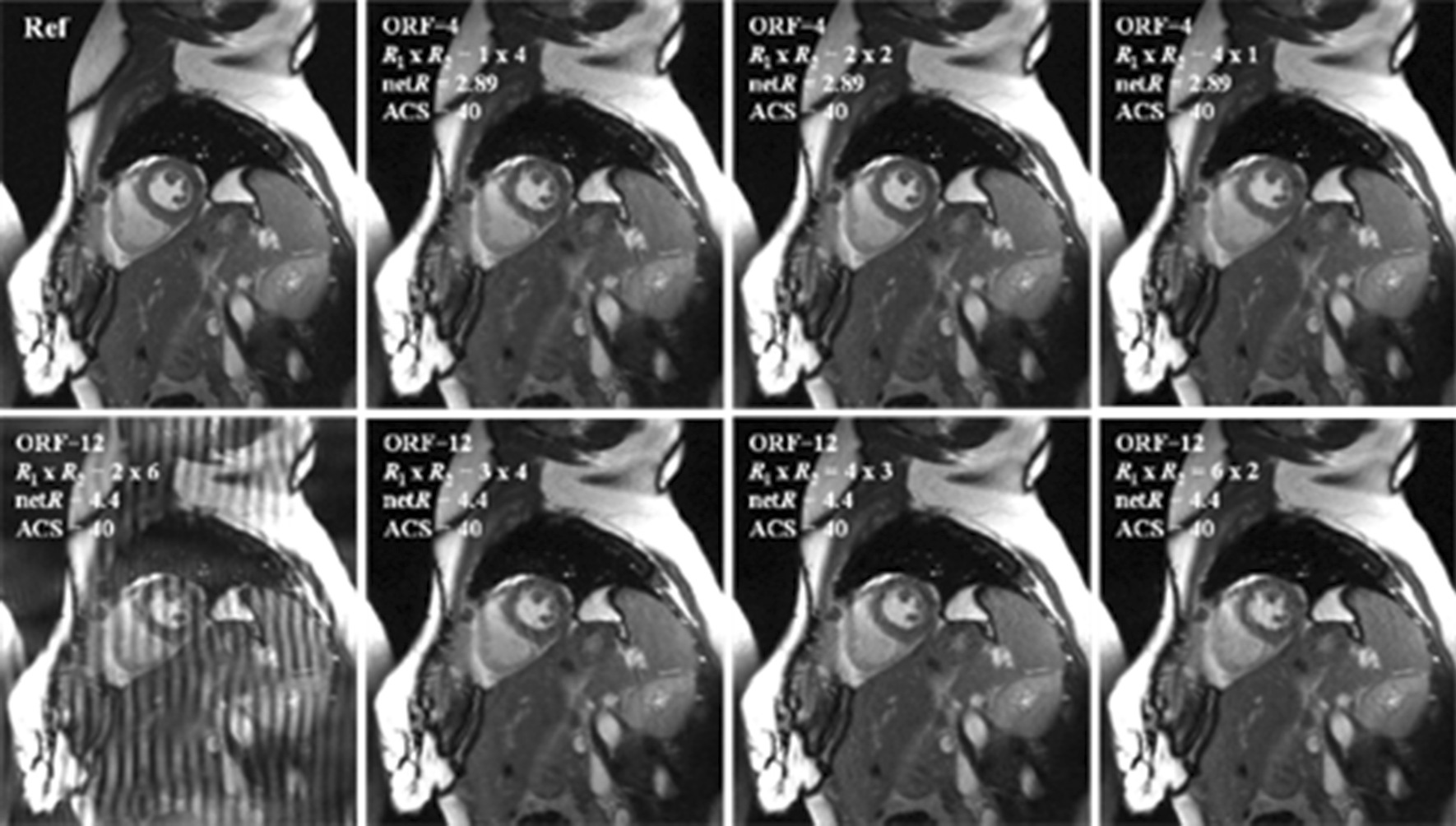
The proposed method of the Data 3 with different combinations of reduction factors

**Fig. 10:**
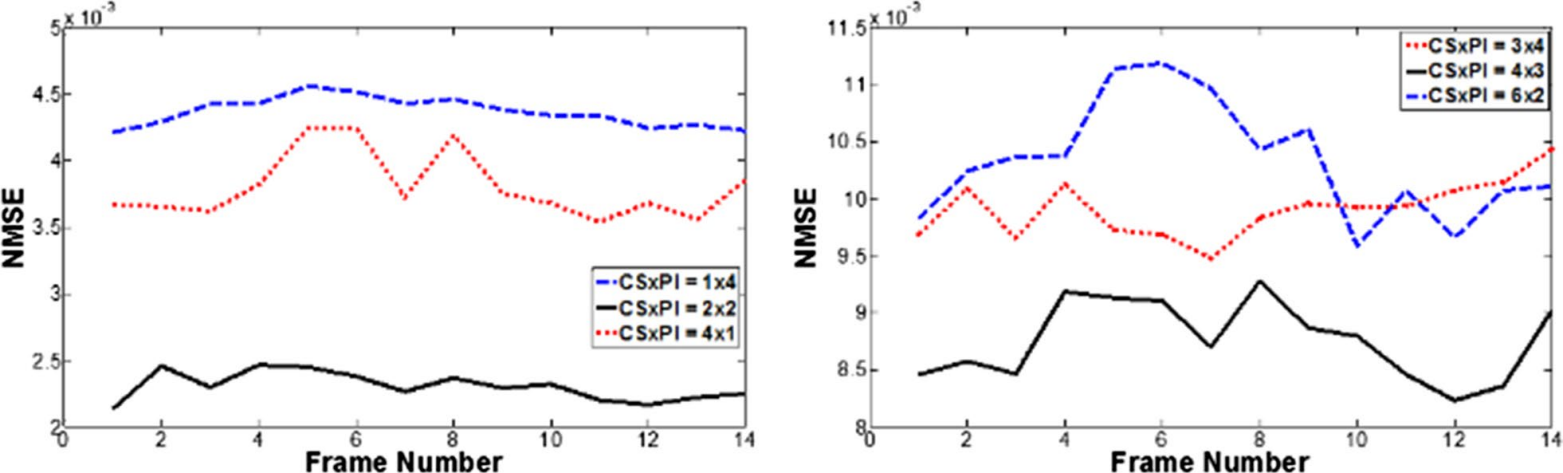
The NMSE plots of the proposed method reconstructions of the Data 3 with different combinations of reduction factors

In CS and PI combinations, the error propagating property [28] has been proven to be critical to reconstruction quality; hence, the choice of the acceleration combination is critical to the final result. Figure 9 presented the comparison of the reconstruction quality of Data 3 with different acceleration combinations for CS and PI at the same net reduction factors *R* = 4 (1 × 4, 2 × 2, 4 × 1) and *R* = 12 (2 × 6, 3 × 4, 4 × 3, 6 × 2) with fixed 40 ACS lines. It can be seen that with a fixed number of ACS lines and outer reduction factor (ORF), a small *R*_1_ will result in lost the de-noised power from CS thus causes large error/noise amplification, especially at high ORF as shown in *R*_1_ × *R*_2_ = 2 × 6; a small *R*_2_, on the other hand, will result in more severe aliasing artifacts (e.g. *R*_1_ × *R*_2_ = 6 × 2). In general, if the acceleration factor is evenly distributed, it is best to keep the acceleration at CS step slightly higher than it at PI step in order to avoid error amplification and propagation. This conclusion can be better appreciated at the NMSE plot as shown in Fig. 10.

### Effect on number of ACS lines

**Fig. 11:**
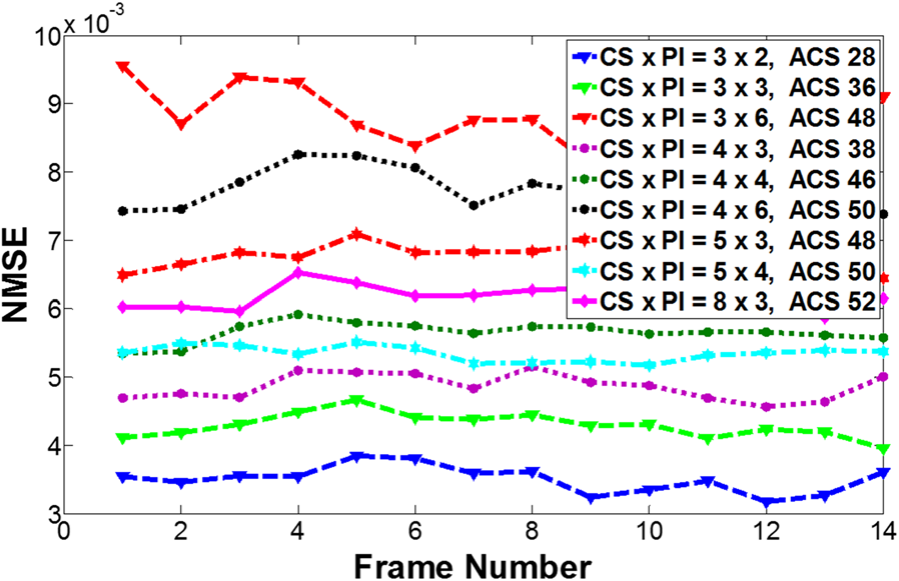
The quality comparison of the proposed method in terms of NMSE of the Data 3 at a fixed net reduction factor of 4.14 with the different combinations of *R*_1_, *R*_2_ and ACS lines

It is very important to carefully choose the number of fully sampled ACS lines as it not only affects the performance of nonlinear GRAPPA [33] but also the key factor of the overall net reduction factor. We conducted an experiment to evaluate reconstruction quality with different combinations of *R*_1_, *R*_2_, and ACS lines at a fixed net reduction factor. Figure 11 shows the comparison of the reconstruction quality in terms of NMSE of the third dataset at a fixed net reduction factor of 4.14. It can be seen that the best performance appears when the reduction factor of CS and PI are both low (*R*_1_ × *R*_2_ = 3 × 2, ACS = 28). However, when the reduction factor is high for PI, even with a large number of ACS, the reconstruction quality was poor as demonstrated in the case of *R*_1_ × *R*_2_ = 3 × 6, ACS = 48; If the reduction factor is evenly distributed and the number of ACS lines is fairly large (*R*_1_ × *R*_2_ = 4 × 3, ACS = 38; *R*_1_ × *R*_2_ = 4 × 4, ACS = 46 and *R*_1_ × *R*_2_ = 5 × 4, ACS = 50), the reconstruction quality is better than that of the higher acceleration at either CS or PI (*R*_1_ × *R*_2_ = 4 × 6, ACS = 50; *R*_1_ × *R*_2_ = 8 × 3, ACS = 52), although they have more ACS lines. This observation is consistent with that been discussed in the previous section. From what has been discussed above, we can get the conclusion that to get the best reconstruction result, the choice of the number of ACS mainly depends on the acceleration factor on PI, when the accelerations for CS and PI are evenly distributed and keep CS slightly higher than PI if necessary.

## Discussion

In this paper, we proposed a novel reconstruction framework that effectively combines compressed sensing and parallel imaging for dynamic MRI reconstruction. With a novel sequential reconstruction strategy together and a tailored sampling scheme, the proposed method was shown to be able to better suppress aliasing artifacts and noise at high accelerations, comparing to the conventional compressed sensing method that incorporated with sensitivity encoding. Although the FOCUSS and nonlinear GRAPPA techniques used in the presented study was previously proposed, the novelty of this study is not to focus on proposing a single reconstruction algorithm, but rather to develop a reconstruction flowchart that can better combine compressed sensing and parallel imaging to ensure the capability to recover high-quality images from maximized acceleration. The reconstruction strategies for each step could easily be replaced by more advanced techniques, i.e. low-rank model [31, 32], dictionary learning [33, 34] for CS reconstruction; E-SPiRiT for the parallel imaging reconstruction [35].

There are many researches that attempt to combine CS and PI [20, 25, 28]. In [28], it used a very similar framework but was limited to the static images. When applying to dynamic MRI, taking the temporal information into account could further reduce the amount of data required for reconstruction. In addition, the inaccurate estimation of the sensitivity information may futher degrade the image quality. In the methods mentioned in Refs. [20] and [25], the sensitivity encoding is incorporated into the Fourier encoding in the data consistency term. Such algorithm exploits the joint sparsity information from all coils, representing the distributed compressed sensing framework. However, such a simple combination is difficult to maximize the accelerations that can be achieved by each individual method because each method has different sampling requirements. In contrary, the proposed framework decouples the acceleration into two sequential steps to maximize the advantages gain from both PI and CS. Experimental results confirmed that the proposed framework can better capture the low contrast cardiac blood flow and preserve more temporal information than the conventional methods. Additionally, employing nonlinear GRAPPA instead of SENSE may also contribute to better suppress noise and remove artifacts. Jung et al. [45] compared the performance of k-t SENSE and k-t GRAPPA applied to cardiac cine and phase-contrast images. They observed that although both methods provide excellent image quality and temporal fidelity for different acceleration factors, k-t GRAPPA demonstrated less spatially varying noise than k-t SENSE. In addition, the sampling trajectory demonstrated in this study uses the combination of random sampling patterns over a uniform undersampling pattern in a Cartesian manner. However, the proposed method could also be applied to non-Cartesian cases, i.e. radial or spiral subsampling, so that many advanced techniques [35–38] could be applied to the proposed framework to achieve better performance.

The proposed method was demonstrated using cardiac cine imaging in this study. However, as it was designed to maximize the acceleration of the data acquisition and improve spatial.-temporal resolution, the proposed method is also expected to benefit other dynamic MR applications, such as dynamic contrast-enhanced MRI and dynamic MR angiography [21, 23]. Nevertheless, it is imperative to choose the best reconstruction strategy for each step based on the specific characteristics of the signal within the application. For instance, in dynamic images whose signal is not periodic, the Fourier transform might not be the best choice to sparsifying the images. In the CS step, Karhunen-Loeve transform or principal component analysis might have better capability to sparse the image thus provides a more accurate k-space reconstruction for the PI step.

While deep-learning techniques for accelerating MR acquisition have gained significant attention in recent years, parallel imaging and compressed sensing continue to play important roles in both clinical practices and research due to their robustness, reproducibility, and repeatability, which have been validated by clinical studies. Although deep learning holds great potential, few studies have evaluated its reliability in certain clinical applications. In light of this, the proposed method still holds great promise for its ability to rapidly adapt to current clinical applications.

The proposed method has higher computational complexity than the conventional compressed sensing based methods with sensitivity encoding. Particularly, in the CS step of the proposed method, the FOCUSS algorithm approximates the solution to the *ℓ*_1_ minimization through iteratively reweighted *ℓ*_2_ minimization. The computational complexity is exactly equivalent to that in k-t FOCUSS [24]; In the PI step, the nonlinear GRAPPA [42] is about 2–5 times that of the conventional GRAPPA [13]. So overall the proposed method has a higher computational complexity than that of k-t FOCUSS [24] or k-t FOCUSS with SENSE [20] due to the nonlinear GRAPPA process. In our current implementation, the total computation time is about 323 s of the proposed method comparing with 19 s that of k-t FOCUSS [24] and 33 s that of k-t FOCUSS with SENSE [20]. In addition to this, this study has limitations. First, the reconstruction techniques used in this study, i.e. k-t FOCUSS and nonlinear GRAPPA, were a bit out of date. Some more advanced or state-of-the-art methods could be applied. This is warranted in future studies. Second, the proposed framework was only demonstrated on a limited number of datasets. A more thorough validation of the proposed framework should be conducted with clinical and patient data before its translation into clinical practice is possible—although the current datasets are adequate as the proof-of-concept for the proposed method.

## Conclusion

We proposed a novel joint framework to sufficiently combines compressed sensing technique with parallel imaging to accelerate dynamic MRI. The proposed method decouples the reconstruction process into two sequential steps: use the CS method to reconstruct a series of aliased dynamic images from the highly undersampled k-space data, and use the nonlinear GRAPPA method to missing k-space data for the original image. The in vivo experiments of cardiac cine imaging suggested that the proposed method can preserve more spatial details and temporal variations of dynamic cardiac images than the state-of-the-art dynamic imaging methods such as the classical k-t FOCUSS method either with or without sensitivity information.

## Data Availability

The datasets used and/or analyzed during the current study available from the corresponding author upon request.
